# Hospital Problems of Buffalo

**Published:** 1910-08

**Authors:** 


### BUFFALO MEDICAL JOURNAL

**A Monthly Review of Medicine and Surgery**

*EDITOR*

WILLIAM WARREN POTTER, M. D.

All communications, whether of a literary or business nature, books for reviewand xchanges, should be addressed to the editor, 238 Delaware Avenue, Buffalo, N.Y.

Vol. Lxvi. AUGUST, 1910. No. 1

### Hospital Problems of Buffalo

FOR more than a year the atmosphere in this city has been redolent with hospital talk, but as yet no plans have been decided upon for the building of the much needed structure. We write in the singular number for we assume that the three hospitals absolutely essential to the city's well being and good name, will be constructed as one plant, and placed under one administration. This, at all events, is the way it should be done, according to our view.

The present needs may be briefly stated as being a hospital for contagious diseases, a hospital for general purposes, and a tuberculosis hospital. To state the question in another way, the city should build a municipal hospital that should make provision for the care of acute and chronic medical and surgical sick and injured; for tuberculosis patients; for those suffering from contagious diseases; and for detention cases, the latter embracing the "pick ups" by the police such as acute alcoholics, suspected insane persons, and other cases to be put under temporary observation.

In short, the city should establish one general hospital with several subdivisions,—as many as may be needed to meet the demands rendered necessary by the modern methods of treatment. Such a hospital should be planned on a scale sufficiently comprehensive to meet present wants and future needs; it should be located sufficiently near the geographic center to be accessible to patients and to physicians; and it should be placed in an environment free from the smoke and noise and turmoil of factories and traffic.

The plan proposed by the Chamber of Commerce through Dr. Charles G. Stockton, chairman of its public health committee, approaches nearer to the proper solution of the problem that confronts the city than any other we have examined. One of the main points to be insisted upon is placing the administration of the hospital under a single head; the responsibility must not be divided and the power vested in the chief must be adequate to provide food and shelter with other necessary comforts for all admitted to its generous care.

But, first of all, we must get the hospital, and get it quickly, too! The good name of Buffalo is challenged, indeed, has been questioned for some time. Shall it be trailed in the dust longer? Shall it be made the victim any longer of the battledore and shuttlecock of small politics? Shall a parsimonious economy longer prevent an appropriation adequate to build a hospital that will reflect credit on the second city of the Empire State?

The summer vacation of the city legislature will prevent taking up the matter again until fall; but when the council and aldermen meet again let their action be short, sharp, incisive; let it be prompt, liberal, and philanthropic! And, moreover, let it be such as will satisfy the pride of a patient, long-suffering, and magnanimous people who ask only justice to the sick and injured, who chance to be stricken within the gates of this great city.

---

Dr. Stephen Smith, one of three physicians who helped to bring about the first training school for nurses in the United States, (*Morning Telegraph*), who is 83 years old and now living at 300 Central Park West, told an interesting story recently of how the system was vindicated by a hunchback clown of the old Standard Theater. In those days Bellevue Hospital was little more than an adjunct to the almshouse.

"Shortly after the first training school was founded I asked Sister Helen if she would give me six of her best nurses for use in my male wards," said Dr. Smith. "Well, you should have seen the faces of the patients when they saw those six pretty, nicely dressed girls enter the wards. Nothing like them had ever been known in a charity hospital in New York. Many people had tried to dissuade me from taking the step, assuring me that it would only make trouble and expose the young women to unpleasant experiences at the hands of the really desperate characters we frequently had foisted on us. But I am happy to say nothing like that ever happened.

"That is to say, I can remember but one such occurrence," he continued, smiling, "and I think I may say it vindicated our system. I remember we had a little dwarf, a hunchback—a clown at the old Standard Theater—sent to us about that time. He had fallen while he was giving some kind of an acrobatic performance on the stage. The man was a jolly, good-hearted little fellow, and he soon became a favorite with everybody in his ward. Just as he was convalescing, as it happened, a tough character from the Five Points district was sent into us, suffering from alcoholism, I imagine.

"When the nurse went to him to find out his needs he started to address a torrent of vile language to her, but he didn't get very far. In a trice the little dwarf had climbed on to his bed and was pounding him for dear life. 'I'll teach you to talk to my nurse like that' he cried out, half-sobbing with rage. 'I'll teach you!' And we actually had to use force to pry them apart."

---

Dr. W. A. Evans, Chicago's Health Commissioner, *The Tribune,* June 8, pleased with the hit that his first series of "healthograms" made a month ago, has issued a new list. The following are samples of the doctor's latest literary effort:

"No spit—no consumption.

"Summer—the time to shun meats and take to vegetables.

"An uncongenial occupation warps the body and withers the soul.

"To relieve worry and sleeplessness take a bath—hot followed by cold.

"When you must drink, drink Adam's ale. Lake Michigan is full of it.

"Dirty milk is better food for bacteria than it is for babies."

---

Dr. Victor D. Lespinasz of the Northwestern University Medical School, Chicago, according to an Associated Press despatch dated May 31, 1910, recently told an alumni audience of physicians that he has discovered a surgical method which may do much to save life and modify suffering. The secret, Dr. Lespinasz explained, consisted in his successful reuniting of severed arteries. Heretofore, Dr. Lespinasz said, physicians have been forced to resort to sewing to obtain the end desired. Stitching was unsuccessful because it too often resulted in the formation of bloodclots, the constriction of the arteries or the enlargement and breaking of them.

Dr. Lespinasz uses rings of magnesium. The severed vessels are joined by these rings or couplers. Magnesium is used because it readily dissolves when the complete union of the severed ends of the arteries has taken place.

The Shah of Persia, who is living at Odessa in a villa placed at his disposal by the Russian Government, has taken up the study of medicine according to a recent press despatch. He is particularly interested in surgery it is reported.

---

In describing the cremation of the body of Dr. Robert Koch, the Baden-Baden correspondent of the Karlsruhe "Zeitung" says: "It was Koch's will, often expressed, that his body be incinerated. When the cortege arrived at the crematory Mozart's 'Ave Verum' was rendered, after which the co-laborer and successor of the scientist, Privy Medical Councillor Gaffky, delivered a eulogy and expressed the sorrow which the whole scientific world shared with those assembled because of the death of one who had so valiantly worked for suffering humanity. Then, to the strains of Mendelssohn's 'Es ist bestimmt in Gottes Rat,' the coffin, heavily laden with flowers, sank out of sight into the devouring flames." —*The Tribune.*

---

According to a press despatch Governor Hughes signed recently a public hospital bill which is of interest to every town, city and village in the state, and which marks a step in advance in hospital development for the State of New York. This bill makes it possible for the common council of a city or the trustees of a village or the governing board of a town, to establish a public general hospital for the care of the sick.

Heretofore it has been necessary for any city, village or town which desired to establish such a hospital to secure a special act on the part of the Legislature, giving them the authority to do so.

Homer Folks, secretary of the State Charities Aid Association, said when the bill was signed by the governor that it was drafted by the association which he represented. Mr. Folks stated that the demand for public hospitals throughout the state is greater today than ever, and that the hospital facilities have fallen far behind the demand.

---

The value of the system of medical defense in vogue in New York is illustrated by the statement that from September, 1900, to January, 1910, the Medical Society of the State of New York has taken charge of two hundred and fifty cases of threatened prosecution of members for alleged malpractice; one hundred and thirty-eight have been actually tried in court, not one finally lost. In one case a verdict was returned against the member, but a new trial has been asked for and a favorable result is expected.

It is announced that Professors Burt G. Wilder, of the department of neurology and vertebrate zoology; Lucien A. Wait, of the department of mathematics, and W. T. Hewitt, of the department of German languages and literature of Cornell University, whose terms of service expire with the academic spring, have been made professors emeritus. They will be retired on pensions from the Carnegie Foundation Fund.

---

The reason doctors do not catch disease (*Chicago Record-Herald*), is because they never think about it. They very seldom take any precautions to secure this amazing immunity, beyond, perhaps, a cold sponge bath regularly, smoking, a pinch of snuff, gargling the throat with some well known disinfectant or washing their hands in an antiseptic solution before and after attending to a patient. A doctor may carry disease from one house to another without contracting it himself. An army surgeon had to cope single-handed with a terrible outbreak of cholera. People were dying around him by the score. When the rush was over the medical man absolutely exhausted, sank onto a bed which a short time previously had been occupied by a bad cholera case, and slept for forty-three hours. Yet he did not contract the complaint, although he had taken no preventive measures. He simply didn't think about it. And that is the whole secret.

---

Chicago's Anti-Spitting Plan could be adopted in Buffalo with propriety. The *Record-Herald* says that with the coöperation of many civic, social and health organisations, thousands of men and women started recently in a concerted movement to further the suppression of spitting anywhere and everywhere.

The first step was taken when cards warning against the dangers of expectorating and the penalties by law attached thereto were issued by Frank E. Wing, superintendent of the Chicago Tuberculosis Institute, to a large number of people working in the interests of the "anti-spitting cause." These "workers" went about their business in the usual way, but whenever they saw anyone spitting would hand a card to the offender, with the polite request that he or she read it.

As a first consignment fifty thousand of the cards were received, and, according to Mr. Wing, the first day's record of distribution bids fair to exhaust the supply in a short while.

---

"I've got an awful case of insomnia," remarked Reid Albee to Jo Paige Smith. "Do you know of a remedy?"

"The best I could advise," returned Smith, "is to eat plentifully and sleep a lot."

